# Clinical, serological and haemodynamic factors associated with poor outcomes in systemic lupus erythematosus-associated pulmonary arterial hypertension: a multicentre retrospective study from Korea

**DOI:** 10.1093/rheumatology/keag148

**Published:** 2026-03-30

**Authors:** Ji-Hyoun Kang, Jiyeol Yoon, Jason Jungsik Song, Hyo-Jin Choi, Seokchan Hong, Chan Hong Jeon, Seung-Geun Lee, Eun Bong Lee, Sang-Hyon Kim, Sung-Eun Choi, Dong-Jin Park, Shin-Seok Lee

**Affiliations:** Division of Rheumatology, Department of Internal Medicine, Chonnam National University Medical School and Hospital, Gwangju, Republic of Korea; Division of Rheumatology, Department of Internal Medicine, Yonsei University College of Medicine, Seoul, Republic of Korea; Division of Rheumatology, Department of Internal Medicine, Yonsei University College of Medicine, Seoul, Republic of Korea; Department of Rheumatology, Gachon University Gil Hospital, Incheon, Republic of Korea; Division of Rheumatology, Department of Internal Medicine, Asan Medical Center, University of Ulsan College of Medicine, Seoul, Republic of Korea; Division of Rheumatology, Department of Internal Medicine, Soonchunhyang University Bucheon Hospital, Bucheon, Republic of Korea; Division of Rheumatology, Department of Internal Medicine, Pusan National University School of Medicine, Pusan National University Hospital, Busan, Republic of Korea; Division of Rheumatology, Department of Internal Medicine, Seoul National University College of Medicine, Seoul, Republic of Korea; Division of Rheumatology, Department of Internal Medicine, Keimyung University School of Medicine, Daegu, Republic of Korea; Division of Rheumatology, Department of Internal Medicine, Chonnam National University Medical School and Hospital, Gwangju, Republic of Korea; Division of Rheumatology, Department of Internal Medicine, Chonnam National University Medical School and Hospital, Gwangju, Republic of Korea; Division of Rheumatology, Department of Internal Medicine, Chonnam National University Medical School and Hospital, Gwangju, Republic of Korea

**Keywords:** pulmonary arterial hypertension, systemic lupus erythematosus, catheterization, right heart system

## Abstract

**Objective:**

To identify factors associated with adverse outcomes in Korean patients with systemic lupus erythematosus-associated pulmonary arterial hypertension (SLE-PAH) confirmed by right heart catheterization (RHC).

**Methods:**

This multicentre retrospective study included 68 patients with SLE-PAH diagnosed by RHC at eight tertiary centres. Baseline demographic, clinical, laboratory and haemodynamic data were collected, along with SLE Disease Activity Index (SLEDAI) scores and PAH-related parameters. Primary endpoint was a composite outcome of worsening PAH symptoms requiring treatment escalation, lung transplantation, death or hospitalization. Univariable and multivariable logistic regression analyses were performed to identify independent predictors.

**Results:**

Of 68 patients, 35 (51.3%) experienced composite outcomes. Compared with those without composite outcomes, patients with composite outcomes had significantly shorter baseline 6-min walk distances (*P* = 0.024), higher tricuspid regurgitation velocity (*P* = 0.032), higher right ventricular systolic pressure (*P* = 0.035), higher mean pulmonary arterial pressure (mPAP) (*P* < 0.001), higher mean physician global assessment scores (*P* = 0.001), higher mean SLEDAI scores (*P* = 0.005) and more frequent use of combination PAH therapy (*P* = 0.033). In multivariable analysis, elevated mPAP (odds ratio (OR) 5.401; 95% confidence interval (CI) 1.129–25.837, *P* = 0.035), higher SLEDAI (OR 5.495; 95% CI 1.041–29.012, *P* = 0.045) and combination PAH therapy (OR 1.695; 95% CI 1.015–3.267, *P* = 0.017) remained independent predictors of composite outcomes.

**Conclusions:**

Elevated mPAP, active lupus and use of combination PAH therapy were independently associated with poor outcomes in SLE-PAH, highlighting the relevance of comprehensive haemodynamic assessment and careful management of lupus activity.

Rheumatology key messages

- Elevated mPAP and higher SLE disease activity predict adverse outcomes in SLE-PAH.
- Combination PAH therapy is associated with poor outcome, reflecting disease severity.

## Introduction

Pulmonary arterial hypertension (PAH) is a rare, progressive and life-threatening disorder characterized by pre-capillary vasculopathy, ultimately leading to right heart failure and death [1]. Connective tissue disease (CTD)-associated PAH is the second most common form of PAH, after idiopathic PAH, and is categorized as Group I PAH [2]. In Western countries, systemic sclerosis (SSc) is the predominant cause of CTD-PAH, whereas studies from Asian countries, including South Korea, have reported that systemic lupus erythematosus (SLE) is a more frequent underlying aetiology of CTD-PAH than SSc [3–5]. This difference in aetiology is thought to reflect racial and geographic variations in the prevalence of CTDs, with Asians, African Americans, Afro-Caribbeans and Hispanic Americans exhibiting a higher prevalence of SLE compared with Caucasians. It also reflects differences in the clinical and serological profiles of SSc patients, since Asian patients with SSc are more likely to express anti-topoisomerase I antibodies and present with diffuse skin involvement and interstitial lung fibrosis, whereas Caucasian patients more commonly exhibit anti-centromere antibodies, which are strongly associated with PAH [6].

SLE patients who develop PAH experience worse outcomes than those without PAH, making PAH one of the leading causes of SLE-related mortality [7, 8]. Thus, early detection and identification of prognostic factors for adverse outcomes, including mortality, are critical for improving management and survival. Several meta-analyses have reported risk factors for the development of PAH in SLE, which may help facilitate early diagnosis and timely initiation of PAH-specific therapies [9–12], and a Chinese group has even proposed a clinical prediction model for estimating the absolute risk of PAH in SLE patients, similar to the DETECT program used in SSc [13].

However, studies focused on prognostic factors for adverse outcomes in established SLE-PAH remain limited. Previous investigations have notable methodological constraints [14], as diagnosis of PAH was often based on echocardiography rather than right heart catheterization (RHC), analyses frequently combined all CTD subtypes rather than evaluating SLE-PAH separately, and in large registries such as COMPERA, the predominance of SSc-PAH cases limited the ability to identify SLE-specific prognostic factors [15, 16]. Our previous study also examined prognostic factors in CTD-PAH as a whole, relying on echocardiographic diagnosis and without separate analysis of SLE-PAH, thereby limiting the generalizability of the findings to this subgroup [17]. To address these gaps, we conducted a multicentre study of Korean patients with SLE-PAH confirmed by RHC to identify clinical, serological, and haemodynamic factors associated with adverse outcomes, including mortality, in this high-risk population.

## Methods

### Study populations

This multicentre, retrospective study was conducted at eight tertiary rheumatology centres across Korea to identify factors for adverse outcomes in SLE patients with PAH. A total of 68 patients were enrolled, all of whom met the 1997 update of the 1982 American College of Rheumatology (ACR) classification criteria for SLE [18]. PAH was diagnosed using RHC, defined as mean pulmonary arterial pressure (mPAP) >20 mmHg, pulmonary arterial wedge pressure (PAWP) ≤15 mmHg and pulmonary vascular resistance (PVR) >2 Wood units [2]. Eligible participants were Korean patients aged ≥18 years of either sex. Patients were excluded if they were diagnosed with PAH without RHC confirmation, or if they had a diagnosis of systemic sclerosis, mixed CTD or an overlap syndrome. Overlap syndromes were operationally defined as fulfilment of established classification criteria for two or more CTDs and/or documentation of an overlap syndrome diagnosis by the treating rheumatologist based on clinical manifestations and serological findings. This study was approved by the Institutional Review Board (IRB) of Chonnam National University Bitgoeul Hospital (IRB No. CNUBH-2024-001). The requirement for written informed consent was waived due to the retrospective design.

### Data collection

We collected data on sociodemographic characteristics, clinical manifestations, laboratory findings and medication use at the time of PAH diagnosis. Clinical features—including malar rash, discoid rash, photosensitivity, oral ulcers, arthritis, pleuritis, pericarditis, renal involvement, neurological disorders and haematologic disorders—were evaluated according to the 1997 ACR revised classification criteria for SLE [18]. Comorbidities such as diabetes mellitus and hypertension were documented. Laboratory parameters included complement levels (C3, C4 and CH50), autoantibodies and antiphospholipid antibodies (lupus anticoagulant, IgG/IgM anti-cardiolipin and IgG/IgM anti-β2 glycoprotein I). Autoantibodies assessed included antinuclear antibody, anti-double-stranded DNA (anti-dsDNA), anti-ribonucleoprotein (anti-RNP), anti-Sm, anti-Ro/SSA, anti-La/SSB, anti-ribosomal P, anti-nucleosome, anti-histone, anti-centromere, anti-Scl-70 and anti-Jo1.

SLE-related outcome measures included the SLE Disease Activity Index 2000 (SLEDAI-2K), Physician’s Global Assessment (PGA), occurrence of SLE flares (mild/moderate or severe) and the Systemic Lupus International Collaborating Clinics (SLICC)/ACR Damage Index (SDI). SLE disease activity was assessed using the SLEDAI-2K and PGA, with the PGA scored on a 0–3 scale [19]. SLE flares were defined according to the Safety of Estrogens in Lupus Erythematosus National Assessment criteria [20]. Irreversible organ damage was evaluated using the SDI, which records cumulative damage from the time of SLE diagnosis, regardless of attribution to lupus [21].

PAH-related outcomes included the 6-min walk distance (6MWD), World Health Organization (WHO) functional class, pulmonary function tests, two-dimensional echocardiography and haemodynamic parameters obtained from RHC. PAH-related symptoms—such as dyspnoea, syncope, dizziness and chest pain—were documented, and serum uric acid, brain natriuretic peptide (BNP) and N-terminal pro-BNP (NT-proBNP) were measured. Pulmonary function testing included assessments of forced vital capacity (FVC) and diffusing capacity for carbon monoxide (DLco). Echocardiographic parameters included right ventricular systolic pressure (RVSP) and tricuspid regurgitation (TR) velocity, while RHC provided measurements of mPAP, PAWP and PVR. To establish the diagnosis of SLE-associated group 1 PAH, alternative aetiologies of pulmonary hypertension were systematically evaluated and excluded. Interstitial lung disease was assessed using high-resolution computed tomography and pulmonary function testing, and patients with ILD considered sufficient to explain pulmonary hypertension were excluded. Chronic thromboembolic pulmonary hypertension was evaluated using ventilation–perfusion scanning and/or computed tomography pulmonary angiography, and affected patients were excluded. Final aetiologic classification was determined based on an integrated clinical, radiologic, functional and haemodynamic assessment at each participating centre.

The primary endpoint was the occurrence of composite outcomes during follow-up, defined as any of the following: worsening PAH symptoms requiring treatment escalation, lung transplantation, death or hospitalization.

### Statistical analysis

Continuous variables were presented as mean ± standard deviation and compared using the Mann–Whitney *U* test or Kruskal–Wallis test, as appropriate. Categorical variables were compared using the χ^2^ test or Fisher’s exact test. Univariable logistic regression analyses were performed to identify potential risk factors for composite outcomes, and variables with *P* < 0.05 were entered into multivariable models. Due to collinearity between TR velocity and mPAP, only mPAP was included in the multivariable analysis. Odds ratios (ORs) with 95% confidence intervals (CIs) were calculated. Analyses were performed using SPSS version 21.0 (SPSS Inc., Chicago, IL, USA).

## Results

Of the 68 patients, 35 (51.3%) developed composite outcomes, whereas 33 (48.5%) did not. Among the 35 patients with composite outcomes, 21 experienced worsening PAH symptoms requiring treatment escalation, 5 underwent lung transplantation, 4 died, and 5 were hospitalized due to aggravation of symptoms during the follow-up. The mean age of the total cohort was 48.4 ± 17.0 years, and 63 patients (92.6%) were female.

Table 1 summarizes the baseline characteristics according to the presence of composite outcomes. Patients with composite outcomes had a significantly shorter symptom duration of SLE than those without (114.5 ± 81.6 vs 117.6 ± 85.4 months, *P* = 0.045). No significant differences were observed between the groups in age, sex, socioeconomic status (education, employment, marital status and insurance) or the prevalence of underlying comorbidities.

**Table 1:** Baseline demographic and clinical characteristics according to the presence of composite outcomes.

Variables	Total (*N* = 68)	With composite outcomes (*N* = 35)	Without composite outcomes (*N* = 33)	*P* value
Age at enrolment, years	48.4 ± 17.0	46.2 ± 14.0	50.9 ± 19.7	0.258
Female (%)	63 (92.6)	33 (94.3)	30 (90.9)	0.926
Symptom duration of SLE, months	115.9 ± 79.6	114.5 ± 81.6	117.6 ± 85.4	0.045
Disease duration of SLE, months	114.0 ± 88.4	114.0 ± 81.6	114.0 ± 96.4	0.999
Disease duration of PAH, months	84.5 ± 56.4	80.9 ± 54.3	88.0 ± 59.1	0.605
Period of education, years	14.1 ± 2.7	13.9 ± 2.6	14.3 ± 2.8	0.675
Health insurance (%)	44 (64.7)	22 (62.9)	22 (66.7)	0.612
Marriage (%)				0.180
Unmarried	20 (29.4)	12 (34.3)	8 (24.2)	
Married	43 (63.2)	20 (57.1)	23 (69.7)	
Divorced	3 (4.4)	3 (8.6)	0 0	
Bereavement	1 (1.5)	0 0	1 (3.0)	
Employed (%)	24 (35.3)	14 (40.0)	10 (30.3)	0.403
Underlying disease (%)				
Diabetes mellitus	2 (2.9)	1 (2.9)	1 (3.0)	0.174
Hypertension	7 (10.3)	4 (11.4)	3 (9.0)	0.790
Hyperlipidaemia	7 (10.3)	5 (14.3)	2 (6.1)	0.579
Osteoporosis	5 (7.4)	3 (8.6)	2 (6.1)	0.933
Peptic ulcer	1 (1.5)	1 (2.9)	0 0	0.428
Depression	1 (1.5)	1 (2.9)	0 0	0.428
Ischaemic heart disease	1 (1.5)	0 0	1 (3.0)	0.200
Congestive heart failure	4 (5.9)	1 (2.9)	3 (9.0)	0.119
Cardiac arrhythmia	4 (5.9)	3 (8.6)	1 (3.0)	0.574
Migraine	3 (4.4)	2 (5.7)	1 (3.0)	0.858
Interstitial lung disease	11 (16.2)	7 (20.0)	4 (12.2)	0.889
Asthma	1 (1.5)	0 0	1 (3.0)	0.200
Hypothyroidism	9 (13.2)	5 (14.3)	4 (12.2)	0.681
Renal failure	5 (7.4)	2 (5.7)	3 (9.0)	0.298
End-stage renal disease	4 (5.9)	1 (2.9)	3 (9.0)	0.119
Solid tumour	4 (5.9)	2 (5.7)	2 (6.1)	0.618
Leukaemia	1 (1.5)	0 0	1 (3.0)	0.200

Unless otherwise indicated, data are shown as mean ± standard deviation.

PAH: pulmonary arterial hypertension; SLE: systemic lupus erythematosus.

Table 2 presents the clinical manifestations and laboratory findings according to the presence of composite outcomes. Anti-β_2_ glycoprotein I IgG positivity was significantly lower in the composite outcome group than in the non-composite group (0% vs 18.2%, *P* = 0.007). Otherwise, there were no statistically significant differences in clinical features—including malar rash, arthritis, serositis and neurologic or renal involvement—nor in the distribution of other autoantibodies (e.g. anti-dsDNA, anti-Sm, anti-SSA/Ro, anti-SSB/La) and complement levels between the groups.

**Table 2:** Clinical manifestations and immunological profiles according to the presence of composite outcomes.

Variables	Total (*N* = 68)	With composite outcomes (*N* = 35)	Without composite outcomes (*N* = 33)	*P* value
Clinical manifestations (%)				
Malar rash	20 (29.4)	10 (28.6)	10 (30.3)	0.876
Discoid rash	3 (4.4)	3 (8.6)	0 0	0.085
Photosensitivity	8 (11.8)	6 (17.1)	2 (6.0)	0.156
Oral ulcer	5 (7.4)	3 (8.6)	2 (6.0)	0.692
Arthritis	32 (47.1)	18 (51.4)	14 (42.4)	0.457
Serositis	20 (29.4)	13 (37.1)	7 (21.2)	0.150
Renal disorder	18 (26.5)	11 (31.4)	7 (21.2)	0.340
Neurologic disorder	4 (5.9)	3 (8.6)	1 (3.0)	0.332
Haematologic disorder	24 (35.3)	9 (25.7)	15 (45.5)	0.089
Raynaud phenomenon	14 (20.6)	9 (25.7)	5 (15.2)	0.282
Digital ulcer	4 (5.9)	3 (8.6)	1 (3.0)	0.332
Interstitial lung disease	11 (16.2)	8 (22.9)	3 (9.1)	0.123
Syncope	3 (4.4)	1 (2.9)	2 (6.0)	0.520
Oedema	13 (19.1)	8 (22.9)	5 (15.2)	0.419
Chest pain	15 (22.1)	6 (17.1)	9 (27.3)	0.314
Dizziness	11 (16.2)	6 (17.1)	5 (15.2)	0.824
Dyspnoea	59 (86.8)	31 (88.6)	28 (84.8)	0.651
WHO functional class (%)				0.067
I	4 (5.9)	0 0	4 (12.1)	
II	30 (44.1)	20 (57.1)	10 (30.3)	
III	23 (33.8)	16 (45.7)	7 (21.2)	
IV	7 (10.3)	4 (11.4)	3 (9.1)	
Autoantibodies (%)				
ANA	66 (97.1)	35 (100.0)	31 (93.9)	0.932
Anti-dsDNA	60 (88.2)	34 (97.1)	26 (78.8)	0.980
Anti-Sm	13 (19.1)	7 (20.0)	6 (18.2)	0.170
Anti-nRNP	27 (39.7)	16 (45.7)	11 (33.3)	0.121
Anti-SSA/Ro	33 (48.5)	17 (48.6)	16 (48.5)	0.782
Anti-SSB/La	16 (23.5)	8 (22.9)	8 (24.2)	0.796
Anti-ribosomal P	3 (4.4)	2 (5.7)	1 (3.0)	0.449
Anti-nucleosome	4 (5.9)	2 (5.7)	2 (6.1)	0.867
Anti-histone	2 (2.9)	1 (2.9)	1 (3.0)	0.910
Anti-centromere	3 (4.4)	2 (5.7)	1 (3.0)	0.449
Anti-Scl70	1 (1.5)	1 (2.9)	0 0	0.295
Anti-Jo1	1 (1.5)	1 (2.9)	0 0	0.295
Lupus anticoagulant	18 (26.5)	8 (22.9)	10 (30.3)	0.474
Anticardiolipin IgG	10 (14.7)	6 (17.1)	4 (12.1)	0.524
Anticardiolipin IgM	7 (10.3)	4 (11.4)	3 (9.1)	0.727
Anti-β_2_ glycoprotein I IgG	6 (8.8)	0 0	6 (18.2)	0.007
Anti-β_2_ glycoprotein I IgM	3 (4.4)	1 (2.9)	2 (6.1)	0.525
Complements (%)				
Low C3	39 (57.4)	23 (65.7)	16 (48.5)	0.420
Low C4	31 (45.6)	20 (57.1)	11 (33.3)	0.123
Low CH50	25 (36.8)	18 (51.4)	7 (21.2)	0.130

ANA: antinuclear antibody; dsDNA: double-stranded DNA; RNP: ribonucleoprotein; Sm: Smith.

Table 3 details the cardiopulmonary and lupus-related clinical parameters according to the presence of composite outcomes. Compared with patients without composite outcomes, those with composite outcomes had a significantly shorter baseline 6MWD (290.5 ± 106.1 vs 367.8 ± 132.2 m, *P* = 0.024), higher TR velocity (4.0 ± 0.7 vs 3.5 ± 0.6 m/s, *P* = 0.032), higher RVSP (68.4 ± 24.5 vs 60.7 ± 22.5 mmHg, *P* = 0.035) and higher mPAP (41.1 ± 13.9 vs 36.0 ± 15.0 mmHg, *P* < 0.001). Additionally, mean PGA (1.3 ± 0.6 vs 0.7 ± 0.5, *P* = 0.001) and mean SLEDAI scores (5.5 ± 3.9 vs 3.9 ± 3.9, *P* = 0.033) were significantly higher in the composite outcome group. Other parameters—including pulmonary function (FVC and DLco), BNP/NT-proBNP, uric acid, SDI scores and flare rates—did not significantly differ between the two groups.

**Table 3:** Cardiopulmonary and SLE-specific clinical parameters according to the presence of composite outcomes.

Variables	Total (*N* = 68)	With composite outcomes (*N* = 35)	Without composite outcomes (*N* = 33)	*P* value
Baseline 6MWD, m	329.2 ± 124.9	290.5 ± 106.1	367.8 ± 132.2	0.024
2D echocardiographic findings				
TR velocity, m/s	3.8 ± 0.7	4.0 ± 0.7	3.5 ± 0.6	0.032
RVSP, mmHg	64.8 ± 23.7	68.4 ± 24.5	60.7 ± 22.5	0.035
Pulmonary haemodynamics				
mPAP, mmHg	38.7 ± 14.6	41.1 ± 13.9	36.0 ± 15.0	<0.001
PAWP, mmHg	12.9 ± 11.0	10.9 ± 5.7	14.7 ± 14.4	0.186
PVR, Wood Unit	3.8 ± 1.6	4.4 ± 1.2	3.1 ± 1.9	0.077
Pulmonary function tests				
FVC, l	4.8 ± 15.9	2.1 ± 0.7	7.3 ± 2.1	0.297
DLco, ml/min/mmHg	9.3 ± 4.2	7.8 ± 3.8	10.7 ± 4.2	0.055
BNP, pg/ml	727.0 ± 1027.1	958.6 ± 1464.1	352.9 ± 525.9	0.605
NT-proBNP, pg/ml	2067.4 ± 2068.9	2091.0 ± 2193.6	2029.2 ± 2700.2	0.956
Uric acid, mg/dl	6.3 ± 2.3	6.6 ± 2.8	6.3 ± 3.1	0.727
SLE related outcomes				
Baseline SLEDAI	6.7 ± 5.9	7.3 ± 5.7	6.2 ± 6.6	0.484
Mean SLEDAI	4.8 ± 3.9	5.5 ± 3.9	3.9 ± 3.9	0.033
Changes in SLEDAI	−3.3 ± 5.6	−3.5 ± 5.7	−3.2 ± 5.6	0.792
Baseline PGA	1.2 ± 0.8	1.4 ± 0.7	0.9 ± 0.7	0.123
Mean PGA	0.9 ± 0.6	1.3 ± 0.6	0.7 ± 0.5	0.001
Changes in PGA	−0.3 ± 0.8	−0.3 ± 0.9	−0.2 ± 0.7	0.755
Flare	0.4 ± 0.2	0.5 ± 0.3	0.3 ± 0.1	0.161
Baseline SDI	1.5 ± 1.2	1.9 ± 1.3	1.1 ± 1.0	0.052
Mean SDI	1.6 ± 1.2	2.0 ± 1.2	1.2 ± 1.1	0.057
Changes in SDI	−0.2 ± 1.4	−0.1 ± 0.6	−0.4 ± 1.9	0.442

Unless otherwise indicated, data are shown as mean ± standard deviation.

6MWD: 6-min walk distance; BNP: B-type natriuretic peptide; DLco: diffusing capacity of the lung for carbon monoxide; FVC: forced vital capacity; mPAP: mean pulmonary arterial pressure; NT-proBNP: N-terminal pro-B-type natriuretic peptide; PAWP: pulmonary artery wedge pressure; PGA: physician global assessment; PVR: pulmonary vascular resistance; RVSP: right ventricular systolic pressure; SDI: Systemic Lupus International Collaborating Clinics/American College of Rheumatology Damage Index; SLE: systemic lupus erythematosus; SLEDAI: systemic lupus erythematosus disease activity index; TR: tricuspid regurgitation.

Table 4 shows the concomitant PAH- and SLE-specific medication use according to the presence of composite outcomes. The composite outcome group was more likely to receive combination PAH-specific therapy (dual or triple) than the non-composite group (monotherapy/dual/triple: 31.4%/28.6%/17.1% vs 39.4%/21.2%/9.1%, *P* = 0.033). Other PAH- and SLE-specific medications did not significantly differ between the two groups.

**Table 4:** Concomitant PAH and SLE medications according to the presence of composite outcomes.

Variables	Total (*N* = 68)	With composite outcomes (*N* = 35)	Without composite outcomes (*N* = 33)	*P* value
Concomitant PAH medications (%)				
Ambrisentan	13 (19.1)	8 (22.9)	5 (15.2)	0.493
Bosentan	6 (8.8)	4 (11.4)	2 (6.1)	0.458
Macitentan	37 (54.4)	21 (60.0)	16 (48.5)	0.411
Selexipag	10 (14.7)	5 (14.3)	5 (15.2)	0.835
Sildenafil	24 (35.3)	14 (40.0)	10 (30.3)	0.456
Treprostinil	4 (5.9)	2 (5.7)	2 (6.1)	0.926
Iloprost	1 (1.5)	1 (2.9)	0 0	0.343
Beraprost	7 (10.3)	4 (11.4)	3 (9.1)	0.184
Type of therapy (%)				0.033
No treatment	18 (26.5)	8 (22.9)	10 (30.3)	
Monotherapy	24 (35.3)	11 (31.4)	13 (39.4)	
Dual combination	17 (25.0)	10 (28.6)	7 (21.2)	
Triple combination	9 (13.2)	6 (17.1)	3 (9.1)	
Concomitant SLE medications (%)				
Hydroxychloroquine	65 (95.6)	34 (97.1)	31 (93.9)	0.520
Daily mean prednisolone dose, mg^a^	5.7 ± 8.2	7.0 ± 10.4	4.2 ± 4.5	0.170
Methylprednisolone IV	16 (23.5)	10 (28.6)	6 (18.2)	0.076
Cyclophosphamide IV	11 (16.2)	6 (17.1)	5 (15.2)	0.867
Azathioprine	20 (29.4)	14 (40.0)	6 (18.2)	0.058
Mycophenolate	22 (32.4)	15 (42.9)	7 (21.2)	0.081
Tacrolimus	10 (14.7)	7 (20.0)	3 (9.1)	0.243
Ciclosporin	4 (5.9)	4 (11.4)	0 0	0.052
Methotrexate	6 (8.8)	3 (8.6)	3 (9.1)	0.541
Rituximab	4 (5.9)	4 (11.4)	0 0	0.052
Belimumab	3 (4.4)	1 (2.9)	2 (6.1)	0.519

a Data are shown as mean ± standard deviation.

IV: intravenous; PAH: pulmonary arterial hypertension; SLE: systemic lupus erythematosus.

Table 5 presents the results of the univariable and multivariable logistic regression analyses for predictors of composite outcomes. In univariable analysis, shorter baseline 6MWD (OR 0.995; 95% CI: 0.990–0.999, *P* = 0.030), higher TR velocity (OR 2.312; 95% CI: 1.045–5.116, *P* = 0.039), higher mPAP (OR 6.225; 95% CI: 1.229–31.531, *P* = 0.027), higher mean SLEDAI (OR 6.314; 95% CI: 2.126–18.753, *P* = 0.035) and use of combination therapy (OR 2.115; 95% CI: 1.220–3.666, *P* = 0.008) were significantly associated with the development of composite outcomes. In multivariable analysis, elevated mPAP (OR 5.401; 95% CI: 1.129–25.837, *P* = 0.035), higher mean SLEDAI (OR 5.495; 95% CI: 1.041–29.012, *P* = 0.045) and the use of combination PAH therapy (OR 1.695; 95% CI: 1.015–3.267, *P* = 0.017) remained independently associated with composite outcomes.

**Table 5:** Univariable and multivariable logistic regression analyses for predictors of composite outcomes.

Variables	Univariable		Multivariable	
	Odds ratio (95% CI)	*P* value	Odds ratio (95% CI)	*P* value
Age	0.983 (0.954–1.013)	0.261		
Female	1.100 (0.146–8.303)	0.926		
Symptom duration	0.999 (0.991–1.006)	0.732		
Anti-β_2_ glycoprotein I IgG	0.513 (0.134–1.970)	0.331		
Baseline 6MWD	0.995 (0.990–0.999)	0.030	0.996 (0.987–1.006)	0.418
TR velocity	2.312 (1.045–5.116)	0.039		
mPAP	6.225 (1.229–31.531)	0.027	5.401 (1.129–25.837)	0.035
Mean PGA	1.109 (0.976–1.262)	0.113		
Mean SLEDAI	6.314 (2.126–18.753)	0.035	5.495 (1.041–29.012)	0.045
Dual/Triple combination	2.115 (1.220–3.666)	0.008	1.695 (1.015–3.267)	0.017

mPAP: mean pulmonary arterial pressure; PGA: physician global assessment; SLEDAI: systemic lupus erythematosus disease activity index; TR: tricuspid regurgitation.

## Discussion

In this multicentre study of Korean patients with SLE-PAH confirmed by RHC, we identified several factors independently associated with composite outcomes, including elevated mPAP, higher SLE disease activity and the use of combination PAH therapy. These findings underscore the association of both haemodynamic severity and underlying lupus activity with adverse outcomes in this high-risk subgroup of CTD-PAH.

Previous studies examining factors associated with adverse outcomes in SLE-PAH have been limited by heterogeneous diagnostic methods, small sample sizes and the inclusion of mixed CTD populations, often dominated by SSc [14]. In large registries, such as REVEAL and COMPERA, the relatively small proportion of SLE-PAH patients precluded robust subgroup analyses [16, 22]. Our study specifically addressed this gap by focusing exclusively on SLE-PAH and enrolling patients with RHC-confirmed diagnoses across eight tertiary centres. Unlike previous studies that assessed CTD-PAH outcomes solely in terms of mortality, we used a composite primary endpoint. Because many patients with SLE-PAH die from infection, unlike those with idiopathic PAH, survival rates alone may not adequately distinguish deaths attributable to PAH from those due to other causes [23]. To more accurately evaluate adverse outcomes in SLE-PAH, we defined a composite endpoint that incorporated multiple clinically relevant events, including mortality, thereby capturing a broader spectrum of disease-related complications.

In the present study, elevated mPAP at diagnosis emerged as a haemodynamic parameter strongly associated with adverse outcomes among the identified outcome-related variables. Early studies of idiopathic PAH, SSc-PAH and SLE-PAH consistently reported mPAP as a significant risk factor for mortality [24, 25]. Similarly, studies of SLE-PAH relying on echocardiography identified elevated pulmonary artery systolic pressure as a prognostic indicator of mortality [7, 26]. However, more recent outcome studies of PAH, including those focusing on SLE-PAH with diagnoses confirmed by RHC, have demonstrated that indices such as right atrial pressure (RAP), cardiac index (CI), and PVR are stronger predictors of mortality than mPAP [2]. For example, in the CSTAR study of SLE-PAH, only CI remained significant as a prognostic factor in multivariate analysis [27]. A subsequent study from the same cohort developed a prognostic model in which PVR, among the various haemodynamic parameters, was incorporated as a mortality-associated risk factor [28]. The reason RAP, CI, and PVR are identified as stronger prognostic indicators than mPAP is that mPAP is determined by the interplay between CI and PVR; thus, RAP, CI and PVR represent more downstream indices. Furthermore, these parameters more accurately reflect right ventricular failure, the primary cause of death in PAH, than mPAP alone. Although the present study may be limited in that it investigated only mPAP as a haemodynamic parameter, it nonetheless emphasizes that RHC-derived haemodynamic variables are essential for predicting adverse outcomes, including mortality, in SLE-PAH. Taken together, our findings underscore the need for comprehensive baseline assessments, including invasive haemodynamic evaluation, as an essential component of risk stratification and management in SLE-PAH.

Notably, we found that higher SLE disease activity, as reflected by elevated SLEDAI scores and PGA, was associated with poor outcomes in this study. While several studies have explored the relationship between SLE disease activity and the development of PAH, no prior studies have specifically investigated the association between disease activity and adverse outcomes, including mortality, in patients with established SLE-PAH. The present study is therefore significant in that it serially assessed disease activity after the onset of PAH in SLE patients and demonstrated its impact on adverse outcomes, including death. Importantly, disease activity was identified as a key risk factor for these outcomes. Histopathological investigations support this finding by showing that, unlike idiopathic PAH, SLE-PAH is characterized by less extensive fibrosis, more pronounced vascular inflammation, and the deposition of autoantibodies and complement in pulmonary arterial walls [1, 29]. Moreover, compared with idiopathic PAH and SSc-PAH, SLE-PAH exhibits a favourable response to immunosuppressive therapy, further suggesting that disease activity plays a pivotal role in shaping disease severity and progression [30]. Taken together, these observations underscore the importance of aggressive control of SLE disease activity to prevent adverse outcomes, including mortality, in patients with SLE-PAH.

Unexpectedly, the use of combination PAH therapy was more frequent in patients who experienced composite outcomes in this study. This association most likely reflects confounding by indication, wherein patients with more severe disease activity or haemodynamic compromise are preferentially selected for intensive treatment. Although this may appear paradoxical, it is better interpreted as treatment escalation in response to more advanced or refractory disease rather than as a causal factor leading to worse outcomes. This interpretation is consistent with findings from prior PAH registries, in which combination therapy has often functioned as a surrogate marker of disease severity [31].

This study has several limitations that should be acknowledged. First, its retrospective design inherently introduces the risk of selection bias and unmeasured confounding, limiting the ability to establish causal relationships between the identified variables and adverse outcomes. Second, although the cohort size is relatively large compared with prior studies of RHC-confirmed SLE-PAH, the absolute number of patients remains modest given the rarity of this condition. Consequently, the statistical power to detect associations with less frequent variables or rare clinical events may be limited, and the possibility of type II error cannot be excluded. Third, the composite endpoint included treatment escalation; therefore, when treatment intensity is also evaluated as a factor associated with outcomes, a degree of circularity and confounding by indication may be introduced. Accordingly, the association between combination PAH therapy and adverse outcomes should be interpreted with caution, as it likely reflects greater disease severity rather than a detrimental treatment effect. Fourth, the use of logistic regression without incorporating time-to-event analyses precluded assessment of outcome timing and longitudinal disease trajectories, thereby limiting interpretation of outcome dynamics. Finally, we did not assess longitudinal changes in haemodynamics using RHC, which may have provided further insights into the dynamic interplay between lupus activity and PAH progression.

In conclusion, this study demonstrates that elevated mPAP, higher SLE disease activity, and the use of combination PAH therapy are independently associated with adverse outcomes in Korean patients with SLE-PAH. These findings highlight the importance of early risk stratification through comprehensive haemodynamic and clinical assessment and emphasize the need for both optimized PAH-targeted therapy and stringent control of systemic lupus activity to improve outcomes in this vulnerable population.

## Data Availability

Data are available upon reasonable request.
